# Incorporation of the emotional indicators of the patient journey into healthcare organization management

**DOI:** 10.1111/hex.13656

**Published:** 2022-11-06

**Authors:** Alfredo Rodríguez‐Fuertes, Pedro Reinares‐Lara, Blanca Garcia‐Henche

**Affiliations:** ^1^ Department of Business Economics Universidad Rey Juan Carlos Madrid Spain; ^2^ Department of Economy and Business Management Universidad de Alcalá Madrid Spain

**Keywords:** emotions, facial expressions analysis, patient experience, patient journey, PREMs

## Abstract

**Background:**

In recent years, attempts have been made to incorporate patients' experiences into healthcare processes, to complement clinical indicators, with what are known as patient‐reported outcome measures (PROMs) and patient‐reported experience measures (PREMs). While the research into PROMs is more developed, the application of PREMs faces some difficulties. The incorporation of emotional indicators into assessments of the experience is an area that remains to be explored.

**Objectives:**

This study proposes a new technique to analyse the emotions experienced by patients during the care process, examines how these emotions influence their satisfaction and propose that if healthcare services focus more on patients' emotions, they can improve the effectiveness of the sector.

**Methods:**

The first, qualitative stage, gathered data from patients to design a patient journey (PJ). The PJ was then reproduced as a video. In a subsequent, quantitative stage, the video was shown to experimental participants, and their emotions were measured through facial expression analysis and a questionnaire.

**Results:**

A new technique to gather emotional data showed that the emotions patients experience do not affect their satisfaction with their clinical care or the physical aspects of the process. However, their emotions did affect their satisfaction with people and organizations.

**Conclusions:**

The importance of the emotional component of patients' experiences was underlined. Therefore, healthcare organizations should take account of this dimension, as well as the cognitive, to increase patient satisfaction and improve their care processes. Understanding the impact of the emotions identified at the subconscious level can help improve the patient experience. A new methodology was applied that may help health professionals to collect emotional data about patients' experiences and to develop PREMs.

**Patient/Public Contribution:**

Patients were involved in all stages of this research. In the exploratory phase, some helped define the touchpoints of the PJ. The data from the subsequent experimental phase were collected from another group, and the emotions they experienced were identified through the analysis of their facial expressions. Based on the results of this study, a working group including patients has been established to work on improvements in the PJ.

## 1 INTRODUCTION

In recent years, there has been an evolution in the management of healthcare organizations, which are now less focused on their professionals, and are more focused on their users and their expectations. This evolution has seen the development of the concept of patient‐centred care. This entails considering both the physical and emotional needs of patients.^1^ In this move from a focus on internal organizational aspects towards a market orientation, and faced with growing demand for healthcare services, an increased understanding of patients' experiences and emotions can improve how services are delivered and increase customer satisfaction. Various authors have emphasized the importance of listening, understanding and learning from the patient experience.^2^, ^3^

In consequence, attempts have been made to develop indicators (other than clinical results) that can allow healthcare managers to incorporate the patient's perspective; thus, patient‐reported outcome measures (PROMs) and patient‐reported experience measures (PREMs) have been introduced. While PROMs are more advanced and are supported by scientifically validated questionnaires, the development of PREMs lags behind and faces challenges such as the difficulty of collecting data on individuals' subjective experiences.^4^

**Figure 1:**
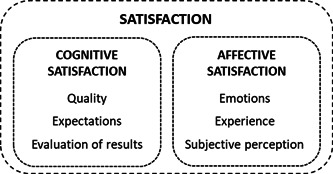
Dimensions of satisfaction

Healthcare providers seek to achieve high levels of patient satisfaction,^5^ one of the most frequently applied healthcare quality indicators. However, although satisfaction is a widely employed concept, there is no consensus on its nature, or on its evaluation.^6^ One of the most accepted theories on the conceptualization of satisfaction proposes that it is a psychological state that can be represented in a double cognitive‐affective dimension,^7^, ^8^ as shown in Figure 1.

The first dimension is cognitive satisfaction, that is, where patients assess the positive and negative aspects of the different components of a service, either by evaluating the perceived outcomes in isolation or by measuring them against a standard or preformed expectations.^6^, ^8^ Cognitive satisfaction with experiences has traditionally been measured through satisfaction questionnaires. The second dimension is the patient's affective evaluation of the experience, which takes into account subjective elements, and captures the emotions generated in the patient by the patient–organization relationship.^9^ With this premise, various studies have demonstrated that, for an objective analysis of satisfaction, account must be taken of both cognitive and affective reactions, since they are different and independently influence the formation and explanation of satisfaction (e.g., Liljander and Strandvik^7^).

Maria Ugolini et al.^10^ highlighted the importance of identifying the emotions that patients experience during the care process. Patients can be in intense emotional states when visiting their doctors. The recognition of, and response to, patients' emotions is related to important healthcare outcomes, including patient satisfaction with the process and adherence to treatment.^5^ Thus, an important aspect of patient‐centred care is the effective recognition of the emotions they go through during medical processes.^11^

Although healthcare organizations have been seen to be increasingly active in improving the emotional experiences of their patients, they still lack a specific approach to addressing these emotions to increase satisfaction levels. Traditionally, healthcare organizations have focused on evaluating the cognitive aspect of satisfaction, and ignored the emotions generated during the care process.^12^ Altringer^13^ highlighted the need for further research into the patient's emotional experience during the care process. Arguably, there is no other service setting in which emotions are more important than in health care. Understanding and managing emotions during the service experience is an important area of research because emotions influence customer perceptions, future intentions and behaviours.^14^

Experience‐based design is a method used to capture the emotional content of patient healthcare experiences and can serve as the foundation for patient‐centred healthcare. As previously noted, an important aspect of patient‐centred care is the effective recognition of the emotions evoked in patients during the healthcare process. Helena Vinagre and Neves^15^ showed just how important positive emotions are for patient satisfaction. Emotions have been shown to be highly predictive in consumer satisfaction models; it has been demonstrated that satisfaction can be influenced by positive and negative emotions. Positive patient experiences have a positive effect on clinical outcomes and cost‐effectiveness. Surveys are commonly used to evaluate satisfaction with care experiences.^9^, ^16^ Nonetheless, while surveys are valid tools for measuring the cognitive component of satisfaction, they have some important limitations. First, surveys have difficulty in assessing the affective component of satisfaction due to the complexity of the respondents' emotions, and in predicting future behaviours^6^, ^17^ ‐ there is evidence that emotions better predict behavioural intentions than do cognitive measures. Second, their inability to evaluate the complete experience, since they generally assess user satisfaction only at one specific moment, although emotions are experienced before, during and after service delivery.^16^ Qualitative research techniques have sometimes been used in combination with quantitative techniques to identify the key determinants of the quality of health services.^7^, ^18^, ^19^ Based on this background, the following study objectives are proposed:

− To validate a methodology that evaluates the affective dimension of patient satisfaction during the care process/patient journey (PJ) and collects data for the development of PREMs.
− To confirm if a relationship exists between the emotions recorded during the PJ and satisfaction measured through a questionnaire.
− To develop proposals for actions to improve care processes in healthcare establishments.

## 2 METHODS

**Figure 2:**
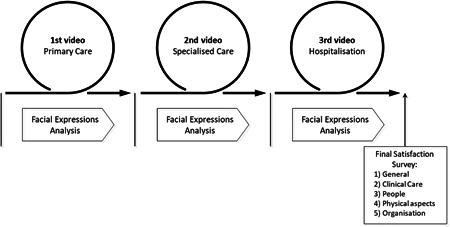
Experimental schema

The present study, undertaken sequentially, used various methods validated in previous studies, to ensure the scientificity of the results.^20^ First, an exploratory phase was carried out using qualitative techniques. Second, an experiment collected neurophysiological data, which was complemented by data collected through a questionnaire (Figure 2). The main methodological novelty was combining traditional data‐gathering techniques with the observation of motor behaviour through facial expression analysis (FEA).^21^, ^22^

In the exploratory phase, the general objective was to identify the emotions generated in patients during a healthcare experience, that is, the PJ, in one of the surgeries most frequently carried out in the Spanish health system, that of hospital‐based inguinal hernia repair. The same process was subsequently examined in the main experiment. The term ‘patient journey’ here refers to ‘the processual and experiential aspects of service processes as seen from the customer viewpoint’.^23^ The PJ journey is here represented graphically as a sequence of patient movements through the care process, showing the interactions between the patient and the various other agents. In addition, this qualitative phase sought to identify patients’ perspectives about the quality of, and their satisfaction with, the experience. In this qualitative stage of the research, the participation of the patients and their companions was essential. First, they described, from their perspective, how the PJ developed, and they helped identify the key points/moments in this particular healthcare experience, and their significance for patients; and, second, they helped the researchers establish a standardized PJ.

Focus group and in‐depth interview techniques were employed in two stages; these had different objectives:

1. To achieve a good understanding of the most important elements of the PJ by defining the most important touchpoints in the care process. Specifically, the profile of the people who participated in this stage was as follows:
  a. Three focus groups, with a total of 21 participants:
    1. Groups 1 and 2: The participants had to have been hospitalized (or had accompanied a hospitalized person) in the previous 3 months.
    2. Group 3: the participants had to have attended a specialized care consultation in the previous 3 months.
  b. Four in‐depth interviews with patients/companions:
    1. Three patients who had been hospitalized in the previous 3 months.
    2. A companion of a person who had been hospitalized in the previous 3 months.
  c. Eight in‐depth interviews were conducted with healthcare professionals and experts in healthcare quality (as carried out in other types of research^24^) to understand the care protocols.

2. Individual in‐depth interviews were conducted with two patients who had undergone hernia surgery in the previous 2 years to review the defined PJ and reach a consensus on the PJ that would be used in the experimental phase.

The guides used in the qualitative techniques were developed based on the objectives established for the exploratory phase of the research. The general objective was to identify the emotions generated in the patients during the healthcare experience. Other objectives were to identify the touchpoints between the patient and the health service provider and to follow the PJ of someone undergoing inguinal hernia surgery.

The objectives of the experimental phase were to obtain quantitative data to identify the emotions experienced by patients during the PJ and to establish their influence on satisfaction. It should be noted that the experiment did not focus only on major phases of the patient experience, rather it examined the complete process, that is, the entire PJ through the different levels of care.

The PJ was recorded on video and tries to show the whole process that the patient goes through, that is, from the appearance of the relevant symptoms until his/her discharge from the hospital. The methodology of collecting facial expression data from participants while they watch videos has previously been used in the analysis of care service processes.^25^, ^26^

The videos were recorded from the patient's perspective. First‐person sequences help immerse the viewer in the scene. So, in the videos, the professionals addressed themselves directly to the camera, as if the patient were in front of them. This means that no patient is featured in the videos.

The recording was divided into the three phases of the patient's experience at each healthcare level (primary care, specialized care and hospitalization) based on the touchpoints previously identified. The division of the process into three independent phases is valid as different types of emotions are identified in each phase (as was verified in the exploratory phase).

The three phases were as follows:

1. Phase 1: Primary care (PC): Prediagnosis.
  - When the patient develops his/her first symptoms and requests an appointment and the first consultation. This took place in the patient's home and in the primary care centre (consultation and appointment area). The participants were the patient and the PC physician.
2. Phase 2: Specialized care (SC): Diagnosis.
  - Took place in the specialized care centre, in the admission zone, and in the consultation rooms of the specialist doctor, nurse and preanaesthetist. The participants were the specialist doctor, a nurse and the anaesthetist.
3. Phase 3: Hospitalization (H) and Discharge.
  - Took place in different areas of the hospital (admission, patient's room and operating theatre), and in the PC consultation room. The people involved were the admission official, a ‘green jacket’, a person responsible for the nexus between the healthcare staff and patients and relatives, a nurse, the doctor who performs the surgery (the same specialist doctor) and the PC doctor.

The recordings, made with the appropriate authorizations, were carried out by a professional audiovisual production team, in a real health facility, with the participation of real health professionals (medical doctors, nurses and ‘green jackets’). The health professionals acted just as they do in real consultations. The facilities depicted were the consultation rooms and other areas in the hospital.

Some 60 people took part in the experiment (mean age: 28.5 years; SD: 2.21; 30 male/30 female), a sample size greater than in other studies that have used the FEA technique.^25^ The universe consisted of individuals between 18 and 65 years. The participants were randomly recruited from a database of representative health service users of all ages. Three conditions were established to participate in the experiment, that is, the patients were users of the public health service, they had not undergone surgery in the previous 24 months and had never undergone inguinal hernia surgery. This meant they were, on average, relatively young. All of them were contacted by email and received financial compensation for their collaboration.

The decision to recruit for the experimental phase only people who had not undergone inguinal hernia surgery and who had no recent hospital experience was made because previous experiences could have influenced their responses; experiences are individual and subjective, and previously undertaken PJs might have conditioned the participants' expectations.

**Figure 3:**
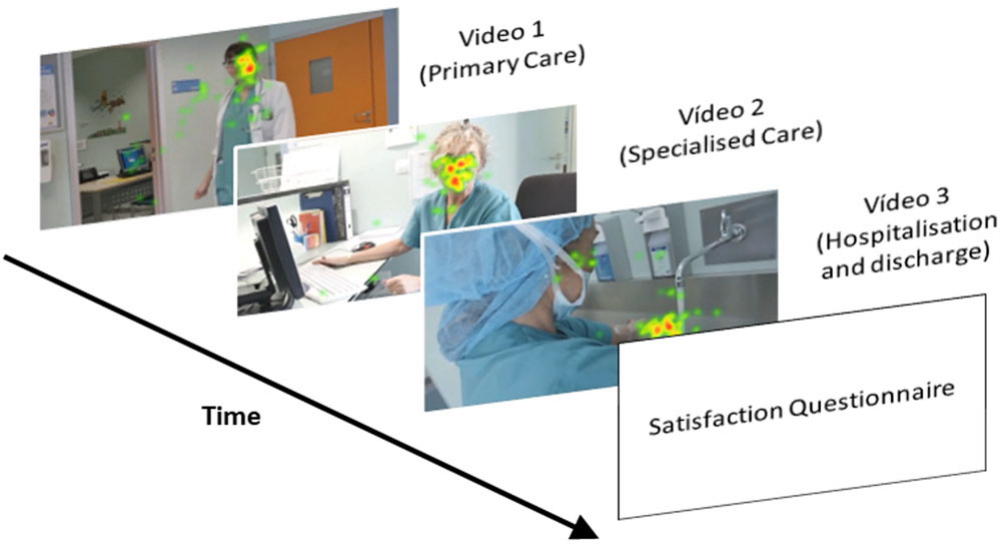
Presentation sequence of the videos used in the experiment

To collect and analyse the emotions that the patients might have experienced during the process the participants' facial expressions were monitored as they watched the videos on a 24‐inch screen (the sequence is shown in Figure 3).

The monitoring was carried out using the Affdex‐Affectiva application. FEA captures a series of basic emotions (joy, anger, disgust, surprise, fear, sadness, contempt), reflected in small movements, which originate in the autonomic nervous system, outside the individual's conscious control, of the facial muscles. The scientific basis of FEA is the correlation found by Ekman and Friesen between emotions and movements of the facial muscles.^27^ The development of FEA has been driven by the use of automated applications capable of identifying facial microexpressions with a duration of less than one‐fifth of a second.

FEA makes it possible to identify the innate primary/basic emotions felt by all people regardless of their individual characteristics. The ability to discriminate between emotions is the main strength of this technique. FEA has received extensive scientific recognition and is used in various research settings. Its validity has been further strengthened by the development of computer applications capable of automatically performing the analysis, which increases the quality and reliability of the information.^28^, ^29^, ^30^

After the viewings, and after the emotions evoked had been recorded, identified and quantified, the participants were asked to assess the experience as if they had been patients. A questionnaire was used to collect information on their degree of satisfaction with the process. The questionnaire asked the participants to provide a general assessment, and an assessment based on the four evaluative dimensions of the patient experience identified by Berry and Carbone: medical care, personal aspects, physical aspects and internal organization.^31^ To evaluate the reliability of the measurement tool Cronbach's alpha index was calculated; this produced a result of 0.852, so internal consistency can be considered satisfactory. The questionnaire allowed a comparison to be made between the self‐report data, that is, cognitive satisfaction, and the neurophysiological data collected by the FEA.

## 3 RESULTS

### 3.1 Qualitative study

The results of this phase of the research showed that the care experience had a high emotional charge for patients and those around them. This focused their demands on those aspects that reduce anxiety and fear. Their expectations were not limited to good technical results, they also expected adequate emotional support throughout the process. The most negative aspects for patients related to difficulties in accessing the healthcare system, for example, postponement of diagnosis and treatment. Once inside the system, their main demands were related to information and treatment.

The PJ became, due to the state of uncertainty induced by concerns about possible negative medical outcomes, a continuous process of searching for trust, through the doctor and the information received in the environment, the tangible aspects, and so forth. Despite the patients' relative state of calm due to their feelings of being under medical supervision, the emotion of fear was continually observed, accentuated at specific times when information was not available, such as when waiting for test results. Negative emotions were observed throughout the care process, particularly anxiety, fear of results and sadness. Positive emotions appeared only with the hope of recovery or with a satisfactory test result.

The analysis of the qualitative information provided a deeper perspective of the patient experience and identified their emotions. We organize the data, below, in three sections, before, during and after hospitalization.

1. Before hospitalization.
  Emotions related to uncertainty/concerns about possible deterioration in one's health situation appear. When accessing the emergency room, the PJ becomes faster and greater tension is felt by the patient and his/her companions. When accessing specialized care, criticisms were expressed about the functioning and organization of the system, particularly about delays, especially in diagnosis, which increases the patient's anxiety.
2. During the hospital stay.
  The hospital stay is a continuous process of seeking trust, through the doctor, the information received, the environment, tangible aspects, and so forth. The stay is a complex phase, very important from the emotional perspective, which produces continuous ups and downs: hope‐optimism, fear‐uncertainty and depression‐pessimism. Initially, the patients experienced a sense of emotional imbalance during the hospitalization process, which reduced only when they became familiar with the environment. Although the patient feels calm being under medical control, the emotion of fear is almost continuous, particularly concentrated in specific phases when no information is available, such as awaiting results. It was observed that as the patient reached a greater emotional balance, and his/her expectations for the results of the healthcare process were met, (s)he became more demanding about comfort aspects, such as noise, food and cleanliness.
  The behaviours of the professionals were regarded positively, that is, in terms of competence, efficiency and information received. Although the doctors' behaviours were usually regarded positively, the lack of adequate communication at some points during the stay generated sadness, loneliness and anger.
  Companions were important during the hospital stay because they provide emotional support to the patient. However, they are more critical of nonmedical care aspects. They demand more information and more comfort during their stay.
3. Following the hospital stay/discharge.
  In general, in the discharge phase, positive emotions of joy and hope were observed, but so were nervousness and fear related to the insecurity felt at leaving the hospital. In this phase, it is very important that the patient is given information (particularly oral) to provide him/her with a feeling of security. While for the hospital the discharge is an administrative process, for the patient it is a very important point at which (s)he needs support and encouragement from the professionals.

From the analysis, it was possible to identify 32 touchpoints/moments that helped build the virtual PJ in the video later used in the research (see Table 1). Finally, the results confirmed the multidimensional nature of patient satisfaction, that is, clinical care, personal treatment and physical and organizational aspects, and these were subsequently incorporated into the survey questions.

**Table 1:** Touchpoints analysed in the patient journey

Stage	Touchpoints
Primary care (PC)	1. Introductory text 2. First symptoms 3. The patient makes an appointment via the internet to see a general practitioner at the health centre 4. Text explaining the patient is on his/her way to the health centre 5. The patient goes to the health centre 6. The patient enters the doctor's consultation room 7. After listening to the patient, the doctor diagnoses an inguinal hernia and refers him/her to a specialist 8. Text explaining the patient is requesting an appointment 9. The patient makes an appointment at the health centre to see the specialist
Specialized care (SC)	10. Text explaining the patient is arriving at the health centre 11. The patient arrives at the SC department for the consultation 12. (S)he enters the specialist's consultation room. The specialist confirms the inguinal hernia diagnosis and refers him/her for tests 13. Text explaining the patient is going for diagnostic tests 14. Diagnostic tests 15. Text explaining the patient is going to the specialist's consultation room 16. The patient goes with the results to the specialist who confirms the need for surgery 17. Text explaining the patient is in the preanaesthesia physician's office 18. Consultation with the preanaesthesia physician
Hospitalization and discharge (H)	19. Text explaining the patient is going to the hospital 20. Arrival at hospital admission desk 21. A ‘green jacket’ talks with the patient 22. The ‘green jacket’ accompanies the patient to the room 23. The ‘green jacket’ shows the patient the room 24. A nurse provides information about the surgical intervention 25. The patient is taken to the operating theatre 26. After entering the operating theatre, the patient is anaesthetized 27. The patient awakes from the anaesthesia 28. Text explaining what happens the next day 29. The physician gives him/her the relevant report and confirms his/her discharge 30. The patient leaves the hospital 31. Text explaining the patient will be going to the primary care consultation in the health centre 32. A few days later, the patient goes to the health centre

### 3.2 Experiment

The results obtained from the FEA allow us to identify the most prevalent emotions in the PJ, ordered from strongest to weakest:

1. Disgust. A reaction to uncomfortable or annoying situations that generate avoidance responses. Disgust registered its highest values at the first moment of the analysis, that is, when the individual was informed of the existence of a health problem. This state was maintained throughout the experience but was accentuated every time the patient entered a new environment to meet a different professional (arriving at the health centre, beginning the consultation, arriving at the hospital, on the way to the operating theatre, in the operating theatre itself and when exiting the hospital).
2. Surprise. Surprise is considered to be a neutral emotion that usually gives way to other emotions. The emotion appeared in unknown environments, such as at the entrance to the healthcare establishment, when entering the consultation room and on the way to the operating theatre. It was also observed at the time of confirmation of the original diagnosis by the specialist doctor, when awakening from the surgical intervention, on discharge and at the last revision consultation in PC.
3. Fear. Fear is caused by threatening situations and is an instinctive defensive reaction to situations of uncertainty where people find it very difficult to predict what will happen next. Fear presented the most frequent oscillations in the PJ and was associated with interactions with healthcare professionals. Thus, fear showed higher values in phase 2, specialized care (in the specialist doctor's office and in the preanaesthesia consultation), and in phase 3, hospitalization (on the way to the room, when the nurse entered the room to explain the process, when waking up after the intervention and when the patient was informed (s)he must return to the primary care centre for a review consultation).
4. Joy. Joy is considered as a positive emotion linked to well‐being. It was observed during the discharge process and when the health professionals looked, and smiled, at the patient.
5. Contempt. Contempt is considered to be an emotional reaction towards a target individual or group that is perceived as morally or socially inferior to oneself. This emotion arose while waiting and during the patient referral process: requesting an appointment, entering the specialist centre, waiting to enter the consultation room, the anaesthesia consultation, and first contact with the person (i.e., the green jacket) who accompanies the patient to his/her room and on the way to the operating theatre.
6. Sadness. Sadness appears in situations of melancholy and discouragement and is associated with a loss of energy. In the PJ it arose when the patient felt helpless. It was noted at times such as admission to the hospital.
7. Anger. Anger can be interpreted as a hostile reaction and a defence mechanism against fear. Although it was less intense than the other emotions, it was detected during the pre‐diagnostic tests, when entering the hospital and on the way to the operating theatre.

**Figure 4:**
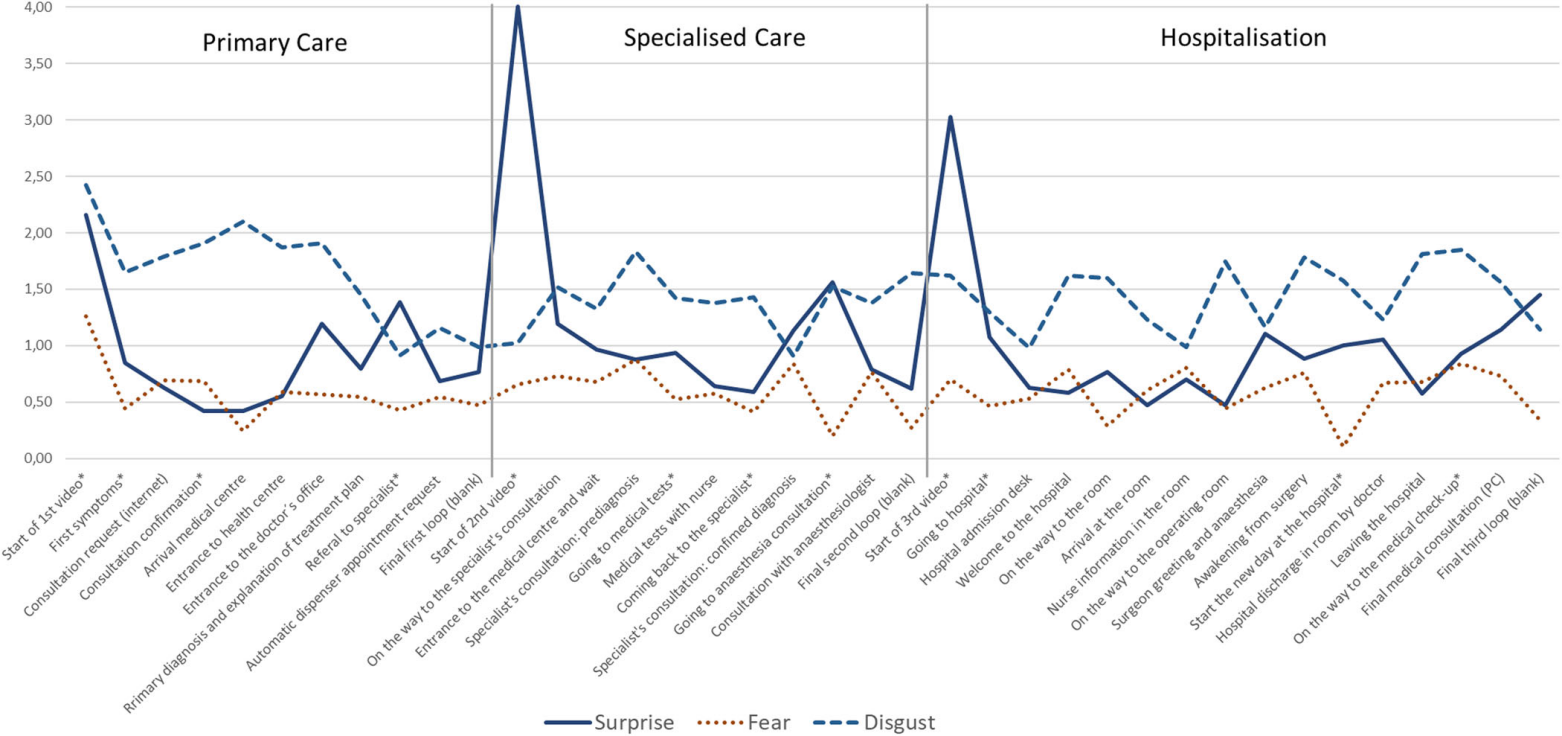
Evolution of the emotions disgust, surprise and fear along the patient journey

The experimental results allowed us to analyse each of the 32 touchpoints in the PJ; greater negative emotion activity was observed in the first phases, and joy appeared in the discharge phase. Figure 4 shows the variations in the three emotions that registered the highest values throughout the PJ, disgust, surprise and fear.

A correlation analysis was performed to determine if any of the basic emotions experienced during the PJ (joy, sadness, anger, contempt, disgust, surprise and fear) influenced the evaluation of satisfaction at the end of the process. The satisfaction results expressed in the questionnaire (general and based on the four dimensions previously discussed) were 8.29/10, the highest rating, for people aspects, 7.78/1.28 for clinical care, 7.39/1.78 for the physical aspects and (6.10/10), the lowest rating, for organization and operations.

Thereafter, an analysis was conducted to investigate whether there were correlations between the satisfaction data collected through the questionnaires, that is, consciously reported by the participants, and the emotion data captured through FEA. The analysis showed only slight correlations between the emotions of contempt and disgust with some factors of cognitive satisfaction subsequently reported by the patients in the questionnaire (Table 2).

**Table 2:** Correlations between emotions and patient satisfaction

	PC		SC		H	
Correlation between	Pearson's correlation	Sig. (bi‐lateral)	Pearson's correlation	Sig. (bi‐lateral)	Pearson's correlation	Sig. (bi‐lateral)
−Contempt and overall satisfaction	−0.353*	0.013	−0.351*	0.013	‐	‐
−Contempt and satisfaction with the organization factor	−0.321*	0.025	−0.365*	0.010	‐	‐
−Disgust and satisfaction with the people factor	‐	‐	−0.386**	0.006	‐	‐

*Note*: Only significant differences are shown.

Abbreviations: H, hospitalization; PC, primary care; SC, specialized care.

* Correlation is significant at 0.01 level (two‐tailed).

** Correlation is significant at 0.001 level (two‐tailed).

As seen in the table, only the contempt felt in the first two phases (PC and SC) had a (slight) influence on the final evaluation of satisfaction. There was a low inverse correlation between contempt and ‘general satisfaction’, both at the end of the PC phase (*r* = −.353 and *p* < .05), and at the end of the SC phase (*r* = −.351 and *p* < .05). A weak inverse correlation was also observed between satisfaction with the ‘organization’, and contempt, at the end of the PC (*r* = −.321, *p* < .05) and SC phases (*r* = −.365, *p* < .05). As a correlation is evident between contempt and ‘overall satisfaction’ in the first two phases, it is possible that, as no correlations with other dimensions exist, this emotion arose due to the organization factor. In addition, a moderate inverse correlation was observed during the SC phase between the emotion disgust and satisfaction with the people factor (*r* = −.386 and *p* < .05).

No correlation was observed between the seven emotions and satisfaction with ‘medical care’. This may be because patients do not question their medical care as they are unable to evaluate it due to a lack of knowledge. Similarly, the emotions recorded during the PJ had no impact on the evaluation of satisfaction with the physical aspects of the experience.

## 4 DISCUSSION

Studies into patient satisfaction have traditionally measured cognitive states, using questionnaires. The present study confirms that the emotional‐affective component is also important, noting that the evaluation of the emotional component of experiences using neurophysiological measures provides substantially different information to that provided by questionnaires.^32^ In addition, the present study confirmed that emotions affect satisfaction, as already demonstrated in previous research,^15^ and the existence of an inverse relationship between negative emotions and satisfaction.^33^ The data analysis also confirmed that negative emotions have a greater impact on satisfaction than do positive emotions.^7^, ^17^ These findings combine to confirm the value of complementing, using neuroscientific methods, the assessment of emotions experienced by patients during healthcare experiences with information collected through conventional satisfaction questionnaires.

The methodology applied in this work opens the door to examinations into new technologies that can collect data on patient experiences and develop PREMs that reflect how patients live their experiences, complementing data collected through questionnaires.

## 5 CONCLUSIONS AND PRACTICAL IMPLICATIONS

Using the FEA technique, it was possible to explore the patient experience and extract data on the emotions they felt. This direct data about the emotions experienced by the patients made it possible to examine specific moments during the PJ.

The most prevalent basic emotions identified in the PJ were the negative emotions of disgust, surprise and fear. Despite the presence of these negative emotions, reported levels of satisfaction were high. Correlations were found between the presence of some emotions and the four dimensions of satisfaction.

This contrast between declared satisfaction and identified emotions confirms the existence of a double dimension in terms of satisfaction, a cognitive element and an affective element.

Given the impact of the two factors organization and people on patients' emotional states, especially in the initial phases, care providers should examine their processes to address patients' feelings of contempt and disgust. Therefore, recommendations are made for these two specific areas:

a. *Improving the organizational aspects of the healthcare system*.
  The impact of organizational aspects on patients' emotions makes it necessary to propose specific actions. Reviews of care protocols should be carried out to ensure they take account of patients' emotions, particularly at those moments that have a greater emotional impact, such as admission and discharge.

In terms of changes to the organizational aspects, institutional efforts should focus on:

1. Generating appropriate emotions when the patient first enters the health centre/designing welcome protocols.
2. Discharge is an emotionally very important moment for the patient, thus efforts must be made to reduce the presence of negative emotions.
3. Given the influence that contempt has on the early stages of the PJ, institutions should take measures to address this emotion by examining their systems of requesting appointments, and delays.
4. As waiting evokes the greatest negative feelings, waiting times to enter consultation rooms should be reduced, or at least the perception of the delays should be addressed by the provision of proactive information systems.
5. Improving the internal orientation of health centres, by providing counters with people who can provide relevant information at entry points, and appropriate signage to help patients locate the appropriate services. Creating ‘high‐resolution consultations’, that is, by scheduling tests and ancillary consultations on the same day. This measure will entail a high level of coordination between services, with the patient being placed at the centre of the organizational effort.

b. *Capabilities of the healthcare professionals*
  The contacts between health personnel and patients are fundamental moments, given that it has been proven that these can generate negative emotions that affect satisfaction. Healthcare professionals must be prepared to respond to the emotional needs of patients. It is proposed that university courses incorporate interpersonal communication skills into their curricula.

## 6 LIMITATIONS

Finally, this work has some limitations. As the experiment focused on a specific patient experience it is not possible to generalize the results. There are disadvantages to assessing the experience using video recordings. When technological advances and resources allow it would be interesting to carry out the experiment in real‐time, using real patient‐healthcare professional interactions in the healthcare service. Also, the emotions observed in the experimental participants should be considered exclusive to that group and, thus, should be treated with caution with a view to generalizing the results.

## CONFLICT OF INTEREST

The authors declare no conflict of interest.

## Data Availability

Data are available on request from the authors.
